# Vaccine Breakthrough Infections of SARS-Cov-2: A Case Report

**DOI:** 10.4314/ejhs.v32i1.20

**Published:** 2022-01

**Authors:** Ramprakash Kaswa

**Affiliations:** 1 Department of Family Medicine and Rural Health, Walter Sisulu University, South Africa

**Keywords:** COVID-19, SARS-CoV-2, mRNA vaccine, Pfizer-BioNTech, vaccine effectiveness

## Abstract

**Background:**

Novel mRNA vaccines provide a high degree of protection against COVID-19 infection, hospitalisation, and death. However, no vaccine claimed 100% effectiveness and it is expected that a small proportion of vaccinated individuals may develop a breakthrough infection. There is a concern relating to the ability of variants to evade vaccine-induced immunity that leads to asymptomatic infection or occasionally progress to disease. The extent of this ability is largely unknown.

**Case Report:**

An 88-year-old male patient was brought to the emergency department on July 25, 2021, with a presentation of a lower respiratory tract infection. He was screened for SARS-CoV-2, and tested positive for the reverse transcription-polymerase chain reaction (RT-PCR) test for SARS-CoV-2. According to the patient, he was diagnosed with benign prostatic hyperplasia (BPH) after a prostate biopsy in 2015 but, at the time of admission, he was not taking any chronic medication. He received the first dose (batch no. FA7812) of the Pfizer-Biotech vaccine on June 8, 2021, and the second dose (batch no. FE2090) on July 20, 2021. He was admitted to the isolation ward with a diagnosis of vaccine breakthrough COVID-19 infection and BPH.

**Conclusion:**

This case report highlights the issue of vaccine breakthrough infections and the potential risk of contracting the COVID-19 disease despite successfully receiving two doses of the Pfizer-BioNTech vaccine six weeks apart.

## Introduction

As of August 15, 2021, the National Institute for Communicable Diseases (NICD) reported more than 2.6 million cases and about 75000 deaths of coronavirus disease 2019 (COVID-19) in South Africa since the first case was confirmed on March 5, 20201. Reducing the transmission of severe acute respiratory syndrome coronavirus-2 (SARS-CoV-2) infection and preventing the disease are the main expectations throughout the world (1). Novel messenger RNA (mRNA) vaccines are fulfilling these expectations but the emergence of new SARS-CoV-2 variants is the biggest threat to their efficacy (2).

South African Health Products Regulatory Authority (SAHPRA) approved Pfizer-BioNTech mRNA vaccine rollout across the country on May 17, 2021, with a two-dose requirement of 3 to 4 weeks apart. The Pfizer-BioNTech vaccine initial clinical trials reported vaccine effectiveness (VE) from 80% after the first dose to 95% after the second dose. Recent studies from the United Kingdom (UK) and Israel have reported a decrease in the VE of the Pfizer-BioNTech vaccine's protection against COVID-19 infection, hospitalisation, and death (2). A study from the UK on VE after two doses was reported at 93.7% and 88.0% against the Alpha and Delta variants respectively (3).

No vaccine provides 100% protection against SARS-CoV-2 infection and a small portion of vaccinated people might have a breakthrough infection(2). Evidence to date among breakthrough infections reported that vaccines reduced the severity of the disease and hospitalisation among patients with the Alpha and Delta variants of SARS-CoV-2(4), To date, no study has reported breakthrough infections among the Pfizer-BioNTech vaccinated population in South Africa. This case report aimed to highlight the fatal case of vaccine breakthrough infection after receiving two doses of the Pfizer-BioNTech vaccine in South Africa.

## Case Report

An 88-year-old male patient was brought to the emergency department on July 25, 2021, with complaints of shortness of breath, headache, cough, and generalised body weakness. He was screened for SARS-CoV-2, and he tested negative on the rapid antigen test. A nasopharyngeal swab for the reverse transcription-polymerase chain reaction (RTPCR) test for SARS-CoV-2 was sent to the National Health Laboratory Services (NHLS).

He presented with blood pressure (BP) of 136/62 millimeters of mercury (mmHg), a pulse of 113 per minute, oxygen saturation of 52% at room air, a body temperature of 37.3°C, and blood sugar at 6.2 mmol/liter. An examination of the respiratory system revealed coarse crackles with decreased air entry in both lung fields and all other systems were within normal limits.

**Figure 1:**
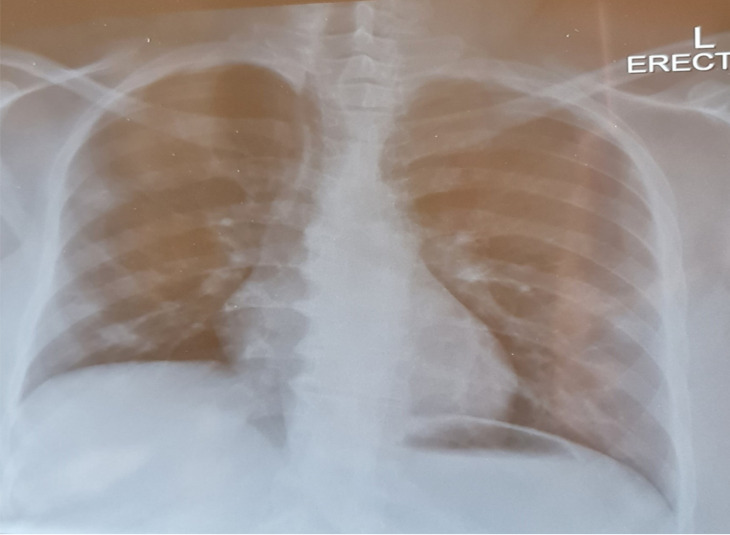
Chest x-ray on day of admission (Source: Created by Author)

He was diagnosed with benign prostatic hyperplasia (BPH) after a prostate biopsy in 2015. He received the first dose (batch no. FA7812) of the Pfizer-BioNTech vaccine on June 8, 2021, and the second dose (batch no. FE2090) on July 20, 2021. He was rehydrated with normal saline and a stat dose of ceftriaxone 1g and admitted to the person under investigation (PUI) ward with a clinical diagnosis of lower respiratory tract infection (LRTI) and BPH. Oxygen saturation was improved from 52% on room air to 90% on a face mask with oxygen at 10 liters per minute flow. The following day, his blood investigation showed haemoglobin at 11.8 mmol/dl, a white cell count of 7.59 × 10^9^/l, platelets at 204 × 10^9^/l, C-reactive protein (CRP) at 214 mg/l, SARS-CoV-2 RT-PCR positive, urea at 12.7 mmol/l, creatinine at 113 µmol/l, and eGFR (CKD-EPI formula) at 51 ml/min/1.73m^2^. A chest x-ray (shown in Figure 1) revealed diffuse bilateral patchy infiltration.

The clinical diagnosis was revised to COVID-19 pneumonia and BPH. He was transferred to the COVID-19 ward and started with dexamethasone 6mg, low molecular weight heparin 40mg, and azithromycin 500 mg. He moved from face mask to high-flow nasal cannula (HFNC) oxygen therapy because he was unable to maintain the target (SpO2 ≥90%) oxygen saturation. He continuously deteriorated despite optimum treatment and died on July 27, 2021.

## Discussion

Although novel mRNA vaccines are highly efficient against SARS-CoV-2 infection, success is dependent upon the effective vaccine immune responses and vaccine coverage of the population. The current evidence indicates that fully vaccinated people are less likely to acquire SARS-CoV-2 (5). However, the risk of rare breakthrough infection in fully vaccinated people cannot be eliminated as long as there is a high community transmission of SARS-CoV-2.

The VE of Pfizer-BioNTech in reducing infection, severe disease, hospitalisation, and death due to COVID-19 has been reported in many recent studies(4). The local experience also supports that vaccines reduce the symptomatic infection and severity of the disease when breakthrough infection occurs after vaccination(3). In this reported case, a severe fatal breakthrough infection occurred after the patient was successfully vaccinated with Pfizer-BioNTech, which is one of the two vaccines authorised in the South African COVID-19 vaccination programme.

To date, the mRNA vaccine has had no significant impact on the Alpha variant, however, the Delta variant has demonstrated a moderate reduction against symptomatic infection (2). These vaccines are still highly effective against severe disease, hospitalisation, and death. Recent studies also reported the immunogenicity is less potent against the Beta variant of SARS-CoV-2 that was first identified in South Africa (4).

Severe disease of COVID-19 infection is associated with a rapid rise of inflammatory response where a large amount of pro-inflammatory cytokine is released. This aggressive immune response of the host towards SARS-CoV-2 is known as “cytokine storm”. Cytokine release is an essential part of the immune system in response to the various pathogen but the exaggerated response of the host can cause worsen the disease process. The cytokine storm is directly correlated with multiorgan failure and high mortality among COVID-19 patients. The rapid deterioration of the clinical conditions of our case might be associated with underlying cytokine storms (1).

In the current case, the patient was 88 years old and might have had less immunogenic response despite the two doses of the mRNA SARS-CoV-2 vaccine. Age could have an impact on vaccine-induced immunogenicity as the elderly do not have a robust immune response like younger people(5). The recent report of the Centers for Disease Control and Prevention (CDC) highlighted the poor immunogenic response among immunocompromised people. In the middle of the growing call for a booster dose of vaccine, the USA approved a third dose of Pfizer-BioNTech for immunocompromised people. At the same time, scientists are observing the ongoing race between immunisation and the natural selection of potential viral escape mutants (4).

Although the novel mRNA vaccine is extremely effective, the risk of rare breakthrough infections cannot be eliminated. This case report cannot differentiate between COVID-19 pneumonia and co-morbidity as an underlying cause of death. Future research is required to evaluate the protection against new emerging variant infections. At present, there are limited data on immune response after successful vaccination among the elderly (above 75 years). The CDC recommended that fully vaccinated people continue to wear a mask in public or indoor settings where a substantial risk of COVID-19 transmission persists(3). Since the country is in the middle of the third wave of the COVID-19 pandemic with a high risk of community transmission, it is advised that fully vaccinated people continue adhering to the nonpharmacological measures to prevent the risk of COVID-19 infection and further transmission.
